# The relationship between healthy eating obsessions, clinical eating disorder, and health anxiety: the dark side of the pursuit of healthy eating

**DOI:** 10.3389/fpsyg.2025.1705064

**Published:** 2025-12-15

**Authors:** Nevin Sanlier, Zeynep Bengisu Ejder, Elif Yildiz Kaya

**Affiliations:** Department of Nutrition and Dietetics School of Health Sciences, Ankara Medipol University, Ankara, Türkiye

**Keywords:** nutrition, orthorexia nervosa, clinical impairment, health anxiety, adult

## Abstract

**Background:**

The obsession with healthy eating, as well as clinical eating disorders and health anxiety, is becoming increasingly common worldwide. Health anxiety, characterised by undue relevance about one’s health and reinforced by perfectionism, is a significant problem requiring intervention in individuals with orthorexia nervosa (ON) and other eating disorders.

**Objective:**

This study was conducted to examine the relationship between orthorexia nervosa, eating disorder and health anxiety in adults.

**Methods:**

A total of 654 people (*M* = 211, *F* = 443) aged 19–50 answered sociodemographic questions and three instruments: the Orthorexia Nervosa Scale (ORTO-R), Clinical Impairment Assessment (CIA) and Short Health Anxiety Inventory (SHAI-18).

**Results:**

Significant positive correlations were observed between ORTO-R and CIA (*r* = 0.461), ORTO-R and SHAI-18 (*r* = 0.364), and CIA and SHAI-18 (*r* = 0.429) (all *p* = 0.000). Regression analyses showed reciprocal associations: higher CIA and SHAI-18 scores were related to higher ORTO-R scores (*β* = 0.350 and *β* = 0.203, respectively; Adjusted *R*^2^ = 0.266), and higher ORTO-R and SHAI-18 scores were related to higher CIA scores (*β* = 0.316 and *β* = 0.262, respectively; Adjusted *R*^2^ = 0.338). Similarly, both ORTO-R and CIA predicted SHAI-18 (*β* = 0.213 and *β* = 0.305, respectively; Adjusted *R*^2^ = 0.228).

**Conclusion:**

In conclusion, it was shown that ON, clinical eating disorder, and health anxiety were significantly related to each other. This finding may contribute to the development of public health communication strategies that promote balanced and evidence-based health behaviors.

## 1 Introduction

The obsession with healthy eating, as well as clinical eating disorders and health anxiety, is becoming increasingly common worldwide. Health anxiety, characterized by undue relevance about one’s health and reinforced by perfectionism, is a significant problem requiring intervention in individuals with orthorexia nervosa (ON) and other eating disorders (Sirri et al., 2020). There is ongoing debate regarding the diagnostic criteria for ON, and the diagnosis has two core criteria: an obsessive focus on “healthy” eating and compulsive behavior and preoccupation that negatively impact clinically (Dunn and Bratman, 2016; Cena et al., 2019). Psychosocial risk factors play a significant role in the emergence and progression of ON and other eating disorders (McComb and Mills, 2019; Strahler, 2020; Rossi et al., 2024), and individuals may exhibit symptoms of anxiety and depression (Strahler et al., 2022). In fact, as conceptualized by Duradoni et al. (2023), in ON, the purity of food is valued above anything else, including deleterious health effects (e.g., an extremely restrictive diet). Researchers suggested that people with ON are anxious about not eating healthy, compulsively plan and prepare healthy meals, and feel superior to others when it comes to choosing healthy foods (Duradoni et al., 2023).

Beliefs about health anxiety are significant predictors of orthorexic symptoms, and it is thought that individuals with high health anxiety may be at higher risk of developing ON (Rossi et al., 2024; Duradoni et al., 2023; Barthels et al., 2021; Chace and Kluck, 2021; Novara et al., 2021a; Greville-Harris et al., 2022). Those with severe anxiety symptoms may exhibit greater levels of emotional eating, uncontrolled eating, and resistance to eating behaviors (Cifuentes et al., 2022). While at first glance, this obsession with healthy eating may seem harmless and can control an individual’s life over time (Zickgraf et al., 2019). While some individuals may perceive their obsession with healthy eating as a positive lifestyle choice (Pontillo et al., 2022; Zagaria et al., 2022; Levin et al., 2023; Tan et al., 2023), for others, healthy eating habits support psychological well-being and when this interest reaches the level of obsession, it can negatively affect mental health (Strahler et al., 2018; Barlow et al., 2024).

Eating disorders can affect an individual’s psychosocial functioning. Individuals’ excessive scrutiny of their body shape and weight can have significant negative effects on their ability to establish and sustain interpersonal relationships (Chace and Kluck, 2021). Some studies have found that individuals with ON have higher anxiety, stress, and lower life satisfaction (Rossi et al., 2024; Strahler et al., 2022; Meier et al., 2020; Novara et al., 2021b). Individuals with eating disorders, such as fear of weight gain and dietary restriction, exhibit significant impairments in clinical and physical health, emotional, and social functioning (Meier et al., 2020; Novara et al., 2021b). However, the relationship between clinical eating disorders and ON remains unclear (Zickgraf et al., 2019). ON is reported to be more common in individuals with a history of eating disorders, restrictive diets, or body dissatisfaction (Novara et al., 2021b). Eating-related clinical disorders are closely related to the rigid, prescriptive, and inflexible structures of orthorexia nervosa (Zickgraf et al., 2019; Zagaria et al., 2022). The potential mediating role of health anxiety in the relationship between an obsession with healthy eating and clinical eating disorders can be explained by the cognitive and emotional processes underlying these variables. Individuals with high health anxiety tend to interpret bodily cues as threatening and develop behavioral control strategies to prevent illness. One of the most common forms of these control strategies is over-structuring one’s diet and focusing on foods perceived as ‘safe’, a process that underlies a preoccupation with healthy eating. Over time, this excessive control can result in a loss of eating flexibility, increased avoidance behaviors and the development of restrictive eating patterns. This, in turn, can result in the emergence or exacerbation of symptoms of a clinical eating disorder. Therefore, it is hypothesised that health anxiety plays a mediating role as an emotional and cognitive trigger in the continuum from healthy eating obsession to clinical disorder.

The boundaries between orthorexia nervosa, health anxiety and eating disorders remain unclear (Strahler et al., 2018). Existing literature emphasizes the need to address excessive health concerns and perfectionistic tendencies in both the prevention and clinical interventions for orthorexic tendencies (Greville-Harris et al., 2022; Barlow et al., 2024; Novara et al., 2021b). In recent years, it has been demonstrated that an obsession with healthy eating is not unique to certain societies but can manifest in a similar way across cultures. Studies conducted in various countries, including Poland, Spain, Germany, China and India, have revealed that this obsession is shaped by social norms, media representations, body ideals and health discourses. Furthermore, longitudinal studies conducted during and after the period of the pandemic indicate that increased health anxiety can reinforce individuals’ tendency to exercise excessive control over their food choices. However, existing literature shows that models addressing obsession with healthy eating, BMI, health anxiety and clinical eating disorder symptoms together are limited. Therefore, this study aims to expand upon and update the existing literature in a cross-cultural context by examining these variables within the same model.

In recent studies, the concept of healthy eating has evolved beyond physical health to encompass psychological and behavioral aspects. This phenomenon, referred to in the literature as ‘healthy eating obsession/orthorexia-like tendencies’, has been found to differ according to various demographic characteristics, particularly age, gender, level of education, and sociocultural factors (Donini et al., 2022; Gleaves et al., 2020). Therefore, this study predicts that obsession with healthy eating will differ based on these variables. Furthermore, research has shown that an obsession with healthy eating can manifest alongside symptoms of clinical eating disorders and is particularly associated with restrictive eating patterns and control-oriented behaviors (Strahler, 2021). It is also recognised that health anxiety can play a regulatory and triggering role in an individual’s eating behaviors and that perceived health threats can lead to excessive efforts to control eating behaviors (Mosewich et al., 2022). Consequently, this study anticipates positive correlations between health anxiety, healthy eating obsession, and clinical eating disorder symptoms.

The aim of this study was to examine the relationship between orthorexia nervosa, clinical eating disorders, and health anxiety in adults and adolescents.

**Figure 1:**
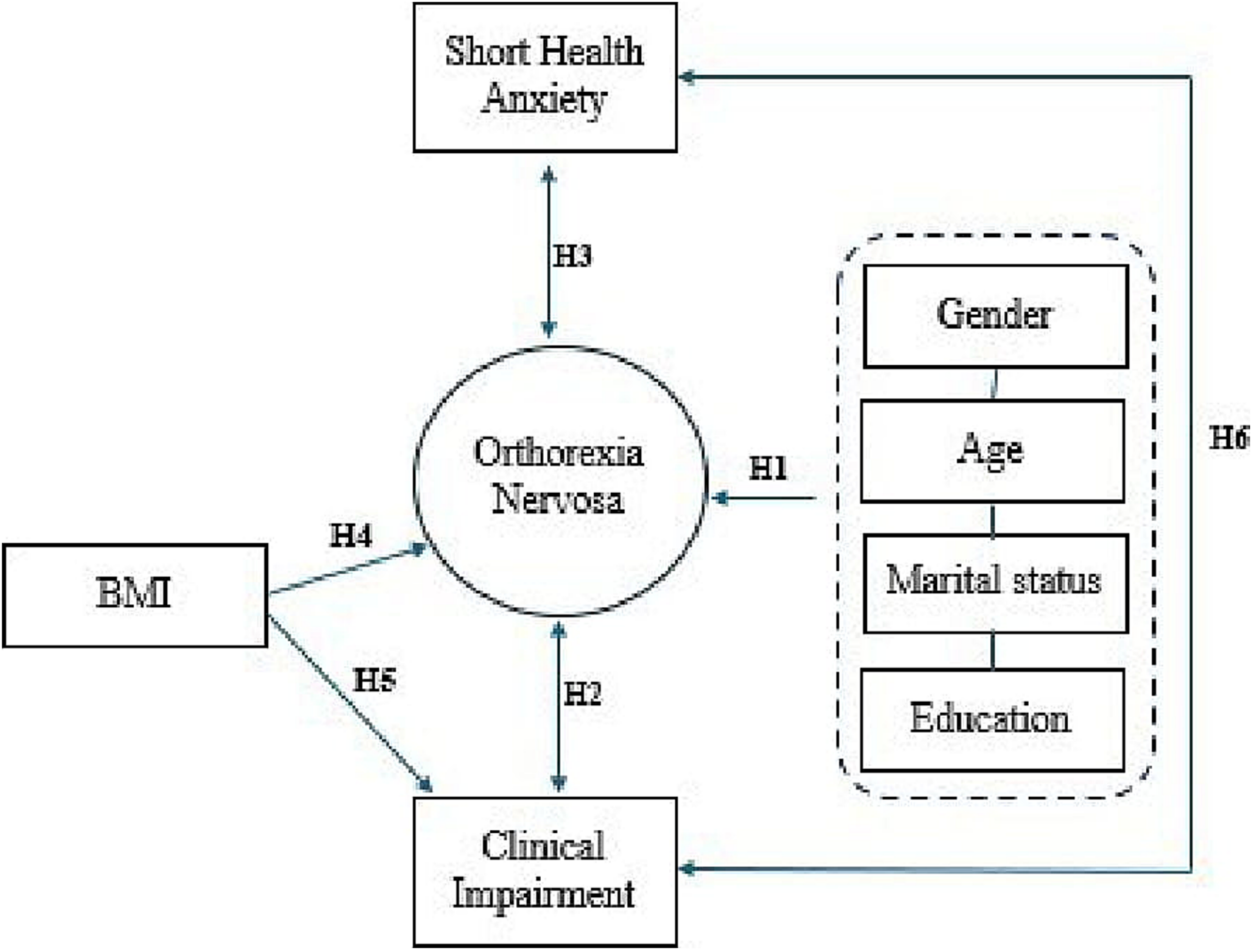
Visual overview of all hypothesis. H1, hypothesis 1; H2, hypothesis 2; H3, hypothesis 3; H4, hypothesis 4; H5, hypothesis 5; H6, hypothesis 6.

The hypotheses of the study are presented in Figure 1.

*Hypothesis 1*: Healthy eating obsession varies by demographic characteristics.

*Hypothesis 2*: Healthy eating obsession is associated with clinical eating disorder.

*Hypothesis 3*: Health anxiety is associated with healthy eating obsession.

*Hypothesis 4*: BMI is associated with healthy eating obsession.

*Hypothesis 5*: BMI is associated with clinical eating disorder.

*Hypothesis 6*: Clinical eating disorder is associated with health anxiety.

## 2 Materials and methods

### 2.1 Study design

The study was a cross-sectional study, and data were collected between March 2023 and January 2024.

### 2.2 Study population

The study included 654 volunteers (211 males, 443 females) aged 19–50.

#### 2.2.1 Inclusion criteria

- Individuals aged >19 and <50.
- Those without chronic diseases.
- Those who agreed to participate in the study.

#### 2.2.2 Exclusion criteria

- Pregnant and breastfeeding individuals.
- Individuals aged <19 and >50.
- Those taking medication for psychological reasons.
- Those with chronic illnesses.
- Those who declined to participate in the study.

### 2.3 Study setting and duration

- Study period: April 2023–December 2023.
- Study location: Data were collected via a questionnaire and face-to-face interviews.

### 2.4 Sample size and sampling method

#### 2.4.1 Sampling size calculation

Before starting the study, a power analysis was conducted using G*Power 3.1.9.7 to determine the number of individuals to be included in the sample. For this purpose, information obtained from similar previous studies (Awad et al., 2021) was used as a reference. The analysis assumed alpha (a) = 0.05, effect size (d) = 0.5, and power (1-b) = 0.95, which indicated a required sample of (*N* = 176). Our final sample (*n* = 654) exceeds this requirement. We chose d = 0.5 based on previous research in the field that reported medium effect sizes (Cohen, 1988).

#### 2.4.2 Sampling strategy

**Figure 2:**
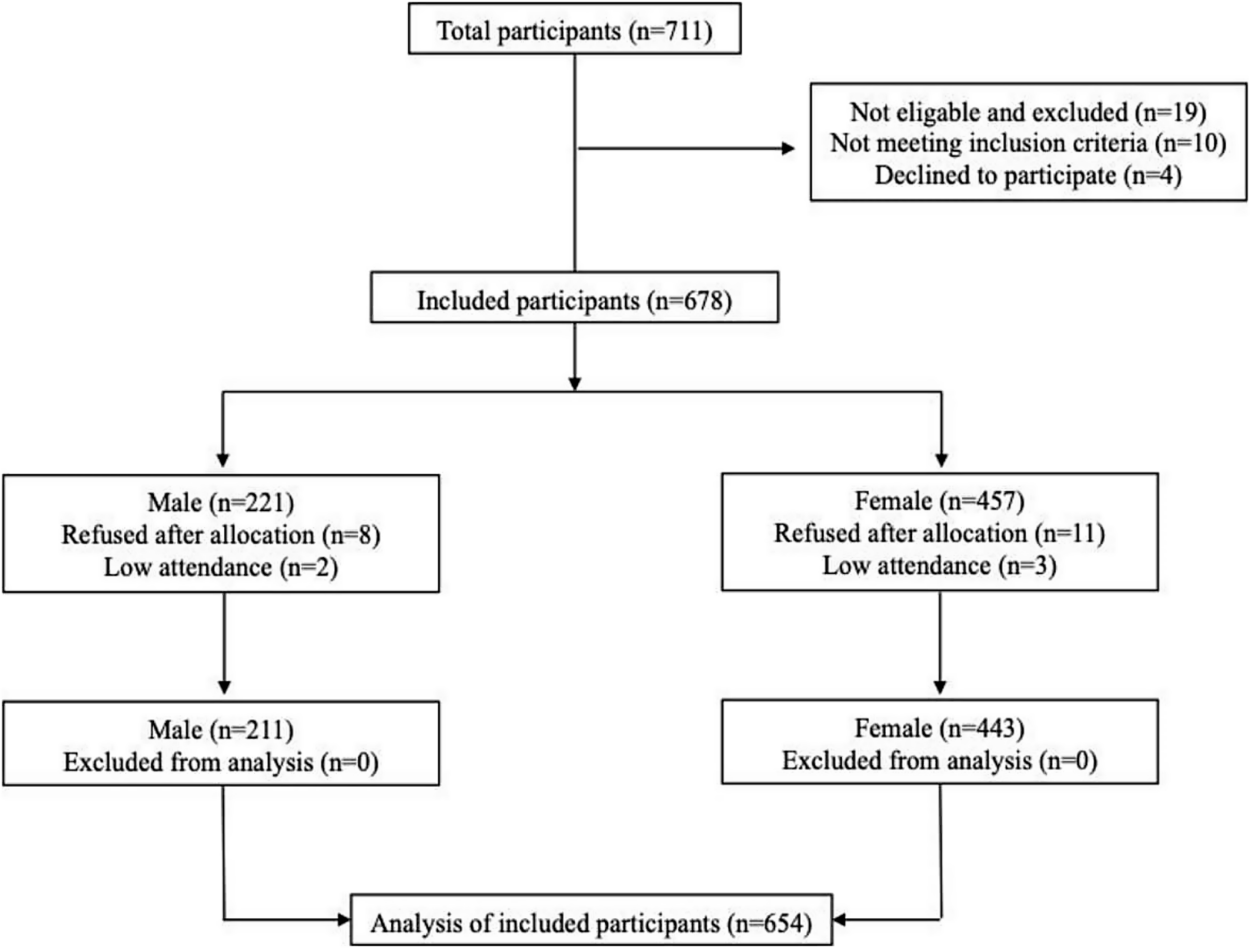
Participant flow chart throughout the study.

The study included 654 volunteers (211 males, 443 females) aged 19–50. A pilot study was initially conducted with 40 participants. Minor modifications were made to address unclear or ineffective questions and wording. The study flowchart is shown in Figure 2. A pilot study was initially conducted with 40 participants. Minor adjustments were made to address unclear or ineffective questions and wording. The study flowchart is shown in Figure 2. Data were collected via a survey and face-to-face interviews. Each participant signed an informed consent form in accordance with the Declaration of Helsinki. Volunteers were seated in a quiet and comfortable position to allow them to easily complete the survey, which took approximately 20 min to complete.

Participants were recruited through a convenience sampling method. This approach was chosen due to the accessibility of the target population and the feasibility of in-person data collection. Although convenience sampling limits the generalizability of the findings, it is widely used in psychological research when probability sampling is not feasible.

### 2.5 Data collection procedures

The data collection tools used in the study are grouped under four main sections;

(1) General information (4 questions).
(2) Orthorexia Nervosa Scale (ORTO-R) (6 items).
(3) Clinical Impairment Assessment (CIA) (16 items).
(4) Short Health Anxiety Inventory (SHAI-18) (18 items).
(5) Anthropometric measurements.

#### 2.5.1 General information

Demographic information of the patients, such as age, education level, gender, and marital status, was collected.

#### 2.5.2 Anthropometric measurements

The researchers measured the body weight and height of all participants. These measurements were then recorded on a questionnaire. Body mass index (BMI) was calculated using the formula body weight (kg)/height (m^2^). Calculations were evaluated according to the World Health Organization’s BMI classification (2025). Participants were categorized as healthy body weight (18.5 ≤ BMI ≤ 24.9 kg/m^2^), overweight, and obese (≥25.0 kg/m^2^) (World Health Organization, 2025). Although BMI was included as a predictor in the present model, this index does not account for body composition or metabolic characteristics, which limits its precision as an indicator of health status.

#### 2.5.3 Orthorexia nervosa scale (ORTO-R)

This scale consists of six items (Rogoza and Donini, 2021). It is a revised version of the 15-item ORTO-15 scale developed by Donini et al. (2005), which has shown an unstable factor structure across different populations. Participants respond using a 5-point Likert-type scale of “never,” “rarely,” “sometimes,” “very often,” and “always.” There is no cut-off point for this scale. Individuals are not classified as having ON based on their ORTO-R scores. Therefore, this scale cannot provide information on prevalence or incidence. Rather, scores obtained from the scale are used for comparison between groups. Although the ORTO-R has shown some psychometric instability and limitations in assessing the prevalence of orthorexia nervosa it was chosen in the present study because it remains the most widely used instrument for assessing orthorexic tendencies.

#### 2.5.4 Clinical impairment assessment (CIA)

This scale is a 16-item self-report measure focusing on the past 28 days designed to measure the severity of psychosocial impairment associated with eating disorder features. It was developed as a measure of functional impairment in the areas of life that are typically affected by eating disorders. These areas include mood, self-concept, cognitive function, job performance and interpersonal function (Bohn and Fairburn, 2008). No study was found on the validity and reliability of the CIA in Turkish. The CIA consists of three subscales to capture clinical impairment in specific field: personal, cognitive and social. Participant responses are rated on a 4-point Likert scale (not at all = 0, a little = 1, a little = 2, a great deal = 3) (Bohn and Fairburn, 2008; Bohn et al., 2008). The CIA overall score is calculated as a intensity index (ranging from 0 to 48). Higher scores indicate bigger force of clinical impairment.

#### 2.5.5 Short health anxiety inventory (SHAI-18)

This inventory comprises 18 items designed to assess health anxiety, regardless of physical health status. These items estimate an individual’s concern about their health, cognizance of somatic sensations and/or changes, and awed consequences of having an illness (Salkovskis et al., 2002). The Turkish validity and reliability of SHAI-18 was made by Aydemir et al. (2013). The scale is assessed using a 4-point Likert scale (never = 0, sometimes = 1, often = 2, almost always = 3). Scores range from 0 to 54 and consist of two subscale scores. Items 1–14 (range 0–42) address health-related anxiety, and items 15–18 (range 0–12) address negative consequences of being ill. Higher scores indicate beliefs about negative consequences of illness and greater health anxiety (Reas et al., 2010).

Although the CIA and SHAI-18 assess distinct constructs, both instruments capture anxiety-related functional impairment, which may partly explain the observed associations.

### 2.6 Data analysis and missing data handling

The data were analyzed using IBM SPSS Statistics 22. Categorical variables were used in the interpretation of the findings. Percentages and distributions were calculated and mean and standard deviation were calculated for quantitative variables. Assessment of data normality was performed through the Shapiro–Wilk test. For normally distributed data, the Independent Samples t-test was used to compare two groups in terms of quantitative variables. The correlation between quantitative variables was analyzed using the Pearson method. Multiple regression analysis was performed for the dependent variables ORTO-R, CIA, and SHAI-18. Prior to conducting multiple regression analyses, assumptions were checked. The residuals were normally distributed, scatterplots suggested homoscedasticity, and collinearity diagnostics indicated no multicollinearity (Tolerance > 0.20; VIF < 5). A significance level of *p* < 0.05 was used in all analyses.

## 3 Ethical considerations

Ethics approval was received for this study from the University Health Sciences Non-Interventional Research Ethics Committee with decision number 37, dated 03/30/2023. The study was carried out in accordance with the Declaration of Helsinki. Participants were informed about the purpose and procedures of the study. Written consent informed consent was obtained from all the participants involved in the study. The study posed no risks to participants, and participants were free to withdraw at any time without any consequences.

## 4 Results

Among the participants, 67.7% were female and 32.3% were male. Most of them (51.8%) were between the ages of 32 and 50, and 53.2% were married. The majority of participants were undergraduates (88.2%), 58.3% (*n* = 381) had a healthy body weight, and 41.7% (*n* = 273) were slightly overweight or obesity (see Table 1).

**Table 1:** Characteristics of the 654 participants included in this study.

Characteristics	*n* (%)
Gender	
Male	211 (32.3)
Female	443 (67.7)
Age (year)	
18–31	315 (48.2)
32–50	339 (51.8)
Marital status	
Married	348 (53.2)
Single	306 (46.8)
Education level	
≤High school	77 (11.8)
Undergraduate	577 (88.2)
BMI	
Healthy body weight (18.5–24.9 kg/m^2^)	381 (58.3)
Slightly overweight or obesity (≥25 kg/m^2^)	273 (41.7)

Cronbach’s alpha coefficients for the scales used in the study, ORTO-R, CIA and SHAI-18, are presented in Table 2. Cronbach’s alpha for ORTO-R was 0.800, CIA was 0.950 and SHAI-18 was 0.910.

**Table 2:** Cronbach’s alpha values of ORTO-R, CIA and SHAI-18 scales.

Scales	Cronbach’s alpha
ORTO-R	0.800
CIA	0.950
SHAI-18	0.910

Table 3 presents the mean (X̄) and standard deviation (SD) scores for ORTO-R, CIA, and SHAI-18, categorized according to the participants’ demographic characteristics. ORTO-R scores were higher in undergraduate (16.5 ± 5.24) (*p* = 0.035) and in those with slightly overweight/obesity (17.4 ± 5.55) (*p* = 0.000). The CIA score was higher in female participants (12.4 ± 11.31) than in male participants (8.5 ± 10.36) (*p* = 0.000), in those in the 18–31 age group (12.9 ± 11.47) than in those in the 32–50 age group (9.52 ± 10.62) (*p* = 0.000), in single participants (13.10 ± 11.63) than in married participants (9.4 ± 10.44) (*p* = 0.000), in undergraduates (11.5 ± 11.24) than in high school graduates or below (8.1 ± 10.02) (*p* = 0.008, 0.000), singles (21.7 ± 10.99) (*p* = 0.016) and undergraduates (20.9 ± 11.00) (*p* = 0.035).

**Table 3:** Arithmetic mean and standard deviation (SD) values of ORTO-R, CIA, and SHAI-18 scores according to participants’ demographic ınformation (*n* = 654).

Demographic information		ORTO-R		CIA		SHAI-18	
		Mean ± SD	*p-*value	Mean ± SD	*p-*value	Mean ± SD	*p-*value
			t		t		t
Gender	Male	15.8 ± 5.44	0.100	8.5 ± 10.36	0.000**	18.6 ± 11.63	0.001^*^
	Female	16.4 ± 5.17	−1.612	12.4 ± 11.31	−4.259	21.6 ± 10.52	−3.267
Age (year)	18–31	16.6 ± 5.39	0.336	12.9 ± 11.47	0.000**	21.9 ± 10.51	0.001^*^
	32–50	16.1 ± 5.16	1.109	9.52 ± 10.62	3.868	19.4 ± 11.27	3.010
Marital status	Married	16.0 ± 5.14	0.146	9.4 ± 10.44	0.000**	19.6 ± 10.90	0.016^*^
	Single	16.7 ± 5.41	1.576	13.10 ± 11.63	4.298	21.7 ± 10.99	2.408
Education level	≤ High school	15.3 ± 5.43	0.035*	8.1 ± 10.02	0.008*	18.1 ± 10.53	0.035^*^
	Undergraduate	16.5 ± 5.24	1.109	11.5 ± 11.24	−2.590	20.9 ± 11.00	−2.115
BMI	Healthy body weight (18.5–24.9 kg/m^2^)	15.6 ± 4.95	0.000**	10.1 ± 10.31	0.029*	20.4 ± 10.63	0.605
	Slightly overweight or obesity (≥ 25 kg/m^2^)	17.4 ± 5.55	−4.258	12.5 ± 12.12	−2.693	20.9 ± 11.46	−0.517

*p < 0.05, **p < 0.001.

**Figure 3:**
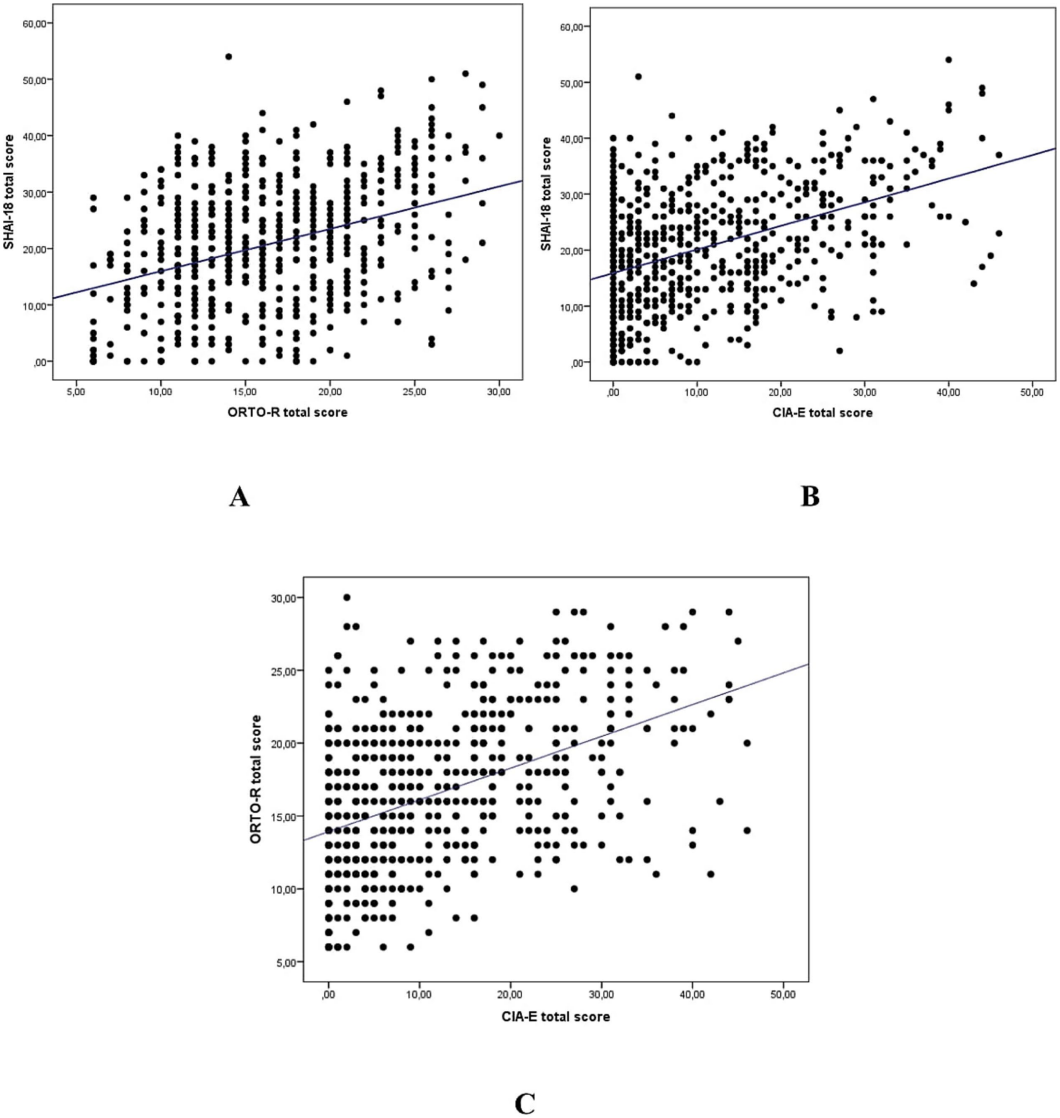
**(A–C)** Relationship between ORTO-R, CIA and SHAI-18 total scores. ORTO-R, orthorexia nervosa scale; CIA, clinical impairment assessment; SHAI-18, short health anxiety inventory-18.

Age showed a small positive correlation with BMI (*r* = 0.384, *p* = 0.000), while it was negatively correlated with CIA (*r* = −0.220, *p* = 0.000) and SHAI-18 (*r* = −0.162, *p* = 0.000). BMI was positively correlated with ORTO-R (*r* = 0.183, *p* = 0.000) and CIA (*r* = 0.122, *p* < 0.01). Significant positive correlations were also observed between ORTO-R and CIA (*r* = 0.461, *p* = 0.000), ORTO-R and SHAI-18 (*r* = 0.364, *p* = 0.000), as well as between CIA and SHAI-18 (*r* = 0.429, *p* = 0.000) (Table 4; Figures 3A–C).

**Table 4:** Correlation analysis between participants’ age, BMI, ORTO-R, CIA, and SHAI-18 scale scores (r).

Variable		2	3	4	5
1. Age (year)	*r*	0.384	−0.059	−0.220	−0.162
	*p*	0.000**	0.129	0.000**	0.000**
2. BMI (kg/m^2^)	*r*	1	0.183	0.122	0.049
	*p*		0.000**	0.002*	0.209
3. ORTO-R	*r*		1	0.461	0.364
	*p*			0.000**	0.000**
4. CIA	*r*			1	0.429
	*p*				0.000**
5. SHAI-18	*r*				1
	*p*				

*p < 0 0.05, **p < 0.001.

Table 5 displays the findings of the multiple linear regression analyses for the participants’ ORTO-R, CIA, and SHAI-18 scores.

**Table 5:** Multiple linear regression analysis of ORTO-R, CIA and SHAI-18.

Variable	*B*	S.E.	*t*	*p*-value	Adj *R*^2^
ORTO-R					
Constant	7.257	1.444	5.027	0.000**	0.266
Gender (female = 1)	0.026	0.429	0.685	0.494	
Age (year)	0.040	0.020	0.827	0.408	
BMI (kg/m^2^)	0.141	0.046	3.639	0.000**	
CIA	0.350	0.018	8.962	0.000**	
SHAI-18	0.203	0.018	5.401	0.000**	
CIA					
Constant	−13.355	2.909	−4.591	0.000**	0.338
Gender (female = 1)	0.087	0.858	2.420	0.016*	
Age (year)	−0.140	0.040	−3.107	0.002*	
BMI (kg/m^2^)	0.154	0.093	4.219	0.000**	
ORTO-R	0.316	0.075	8.962	0.000**	
SHAI-18	0.262	0.036	7.495	0.000**	
SHAI-18					
Constant	9.870	3.116	3.166	0.002*	0.228
Gender (female = 1)	0.041	0.914	1.059	0.290	
Age (year)	−0.088	0.043	−1.805	0.072	
BMI (kg/m^2^)	0.012	0.100	0.291	0.771	
ORTO-R	0.213	0.082	5.401	0.000**	
CIA	0.305	0.040	7.495	0.000**	

*B*, Standardized beta coefficients; SE, Standard error; *t*, Independent samples *t*-test, *p < 0.05, ** *p* < 0.001, Adj *R*^2^, Adjusted coefficient of determination, Gender is coded as 0–1.

Increases in BMI (standardized *β* = 0.141, *p* = 0.000, Adjusted *R*^2^ = 0.266), CIA (standardized *β* = 0.350, *p* = 0.000, Adjusted *R*^2^ = 0.266), and SHAI-18 (standardized *β* = 0.203, *p* = 0.000, Adjusted *R*^2^ = 0.266) were associated with increases in ORTO-R. Decreases in female gender (standardized *β* = 0.087, *p* = 0.016, Adjusted *R*^2^ = 0.338) and age (standardized *β* = −0.140, *p* = 0.002, Adjusted *R*^2^ = 0.338) and increases in BMI (standardized *β* = 0.154, *p* = 0.000, Adjusted *R*^2^ = 0.338), ORTO-R (standardized *β* = 0.316, *p* = 0.000, Adjusted *R*^2^ = 0.338) and SHAI-18 (standardized *β* = 0.262, *p* = 0.000, Adjusted *R*^2^ = 0.338) were associated with increases in CIA. Increases in ORTO-R (standardized β = 0.213, *p* = 0.000, Adjusted *R*^2^ = 0.228) and CIA (standardized *β* = 0.305, *p* = 0.000, Adjusted *R*^2^ = 0.228) were associated with increases in SHAI-18.

## 5 Discussion

Behaviors associated with ON may result in emotional, cognitive, and social outcomes that adversely affect individuals’ daily functioning. Eliminating foods perceived as “unhealthy” may lead to nutrient deficiencies and weight loss Although ON and healthy eating may appear alike at first glance, ON is characterized by rigid dietary rules, whereas healthy eating promotes flexibility and diversity in food choices. In healthy eating, occasional indulgence is accepted without feelings of guilt, whereas ON is marked by adverse emotional responses when one strays from self-imposed dietary standards (Cena et al., 2019; Mitrofanova et al., 2025). An excessive focus on healthy eating, in combination with psychological characteristics like perfectionism (Barlow et al., 2024; Novara et al., 2021b), is consistently associated with increased health anxiety and eating disorder symptoms (Levin et al., 2023; Tan et al., 2023; Donini et al., 2022; Yılmaz and Dundar, 2022).

The relationship between orthorexia and gender is inconsistent. While some studies report no significant gender difference (McComb and Mills, 2019; Barlow et al., 2024; Yılmaz and Dundar, 2022), one study also reports that orthorexia symptoms may be more common in women (Zagaria et al., 2023). It has also been reported that tendencies toward healthy eating are similar between men and women, but the tendency toward healthy eating is more pathological in women (Strahler et al., 2022). There are also studies reporting higher orthorexic tendencies in women, but no significant gender effect or a higher risk in men with higher BMI (Zagaria et al., 2023; Hallit et al., 2021; Sanlier et al., 2016; Oberle et al., 2017). In the current study, ORTO-R did not differ by gender (*p* > 0.05) (Table 3). Clinical eating disorders are linked to a higher risk among women (Gramaglia et al., 2019). In this study, the CIA score was found to be higher in female participants (12.4 ± 11.31) (*p* < 0.001) (Table 3). Health-related anxiety is reported to be twice as common in women as in men (Jahrami et al., 2019). In the present study, female had significantly higher SHAI-18 scores (21.6 ± 10.52) compared to male (18.6 ± 11.63) (*p* < 0.05) (Table 3). Conversely, another study reported no statistically significant gender difference in SHAI-18 scores (Kris-Etherton et al., 2021). This difference observed between men and women may vary according to social roles, suggesting that in some traditional societies, men are not as obsessed with body weight as women.

Young people’s nutritional sensitivities may make them particularly susceptible to ON due to the frequency of eating disorders (Pontillo et al., 2022; Alshayea, 2020). Studies have indicated that eating disorders are most common between the ages of 12–35 (Barlow et al., 2024; Zickgraf and Barrada, 2022; de Vos et al., 2017). In contrast to this result, another study reported no significant age-related difference in ON prevalence (Luck-Sikorski et al., 2019). In the present study, ON was not significantly related to age (*p* > 0.05) (Table 3). Clinical eating disorders are linked to a heightened risk among young individuals (Reynolds, 2018). Findings of another study revealed no age-related variation in clinical eating disorders among young men and women (Argyrides et al., 2020). In this study, participants aged 18–31 had higher CIA scores (12.9 ± 11.47) (*p* < 0.001) (Table 3). Although no difference was found in the SHAI-18 score according to age in another study (He et al., 2022), this study found it to be higher in the 18–31 age group (21.9 ± 10.51) (*p* < 0.05) (Table 3). This may suggest that young age is a factor that increases health anxiety and that young people are more sensitive to these issues (Arnáez et al., 2019).

The impact of marital status is not emphasized or remains secondary in prior studies (McComb and Mills, 2019; Albery et al., 2022; Escolar-Llamazares et al., 2023; Witaszek et al., 2023). In this study, single individuals had higher CIA (13.10 ± 11.63) and SHAI-18 scores (21.7 ± 10.99) than married individuals (Table 3). Another study reported that individuals with higher education levels have more knowledge about a healthy lifestyle and a higher prevalence of ON (Reivan Ortiz et al., 2025). In this study, the ORTO-R score was found to be higher in university graduates (16.5 ± 5.24) (*p* < 0.001) (Table 3). Clinical eating disorders are associated with an increased risk, particularly among university students (He et al., 2022). This study also found that it was higher in university graduates (11.5 ± 11.24) (*p* < 0.05) (Table 3). In this study, the SHAI-18 score was found to be higher in university graduates (20.9 ± 11.00) (*p* < 0.05) (Table 3), suggesting that education level and knowledge accumulation may lead to an increase in health anxiety. Another study reported that university graduates were more anxious about health-related information (Gkiouleka et al., 2022).

The study conducted by Łucka et al. (2025) on two cohorts of young adults in Poland demonstrated that orthorexic tendencies co-occur with anxiety and disordered eating symptoms and vary substantially across different socio-cultural contexts. These findings support the view that ON is a heterogeneous construct and highlight the importance of considering demographic and cultural factors when interpreting orthorexia-related outcomes.

It is thought that individuals with high BMI may be more prone to clinical impairment by causing deterioration in physical functioning and, due to the limitations caused by this condition, deterioration in social functioning (Myrick and Willoughby, 2019). Studies support a positive relationship between increased BMI and ON (Zickgraf and Barrada, 2022; Sahlan et al., 2022). It is thought that the deterioration in body image experienced by obese individuals may make them more obsessed with health. Furthermore, some studies have found no relationship between BMI and ON (Reynolds, 2018; Abdullah et al., 2020). Orthorexia can sometimes occur as a distinct pattern without body image concerns (Barlow et al., 2024; Zickgraf and Barrada, 2022; Chard et al., 2019). Clinical eating disorders are associated with an increased risk of developing high BMI (He et al., 2022). Similar studies have also associated higher BMI with higher CIA, suggesting that increased BMI brings with it many undesirable health risks (Reivan Ortiz et al., 2025; Łucka et al., 2024). One study found that BMI is not a simple indicator of weight but rather a clinical risk indicator (Dudzikowska et al., 2025). In this study, BMI was positively correlated with ORTO-R (*r* = 0.183, *p* < 0.001) and CIA (*r* = 0.122, *p* < 0.05) (*p* < 0.001) (Table 4).

According to Chace and Kluck (2021), orthorexia symptoms were significantly and positively associated with health anxiety, which emerged as an important predictor of the condition. Levin et al. (2023) observed a stronger association between orthorexia subprofiles, health anxiety, and BMI among university students. This suggests that the relationship between ON and health anxiety may be moderate, and that health preoccupation may lead to orthorexic behaviors beyond those seen in traditional eating disorders (Strahler et al., 2022; Barlow et al., 2024; Novara et al., 2021b). Studies support the view that ON is more closely related to eating disorders than obsessive-compulsive disorder. However, the moderate strength of these associations suggests that ON may be a distinct clinical entity (Novara et al., 2021b; Vaz et al., 2020). Another study found strong associations between orthorexia and health anxiety (Kris-Etherton et al., 2021), and the effect of SHAI-18 on ORTO-R was similar to this study (*p* < 0.001) (Table 5).

Individuals may feel superior to others by comparing their own lifestyles and eating habits with those of others through perfectionist behaviors related to healthy eating (Atchison and Zickgraf, 2022). This can lead to an unhealthy mindset based on healthy eating habits (Argyrides et al., 2020). Generally, when the pursuit of healthy eating becomes rigid and obsessive, leading to significant health problems and influenced by factors such as anxiety, perfectionism, and health controllability, a “dark side” can occur (Strahler et al., 2022; Chace and Kluck, 2021; Greville-Harris et al., 2022; Barlow et al., 2024).

This study demonstrates that an obsession with healthy eating, health anxiety and symptoms of an eating disorder may be linked through shared processes of anxiety and control. As health anxiety increases, individuals may be more likely to engage in heightened regulation and restriction of their eating behaviors in an effort to “protect their health.” This pattern may be associated with higher levels of orthorexic tendencies, which in turn are linked to elevated eating-disorder–related symptoms. In the Turkish context, societal expectations regarding body image and the social significance of sharing food can exacerbate the emotional impact of ON. It should also be noted that ON has behavioral, cognitive and emotional dimensions. From a clinical perspective, early screening for ON in individuals with high health anxiety is recommended. From a public health perspective, nutritional messages should emphasise flexibility and balance.

These results emphasize the connection between orthorexic behaviors and clinical eating disorders in relation to health anxiety. Findings from the current study suggest that health anxiety is associated with orthorexic tendencies as well as risk for clinical dysfunction related to eating behaviors.

In addition to the observed associations between health anxiety, orthorexic tendencies, and eating-disorder–related impairment, several psychological mechanisms may help explain these relationships. Elevated health anxiety may be linked to rigid, perfectionistic standards around “clean” eating, increased cognitive control over food choices, and difficulties regulating emotions factors that can reinforce orthorexic and disordered eating patterns. Impulsivity may further contribute to fluctuations between rigid restriction and loss of control (Strahler et al., 2022; Barlow et al., 2024; Sanseverino et al., 2025). Although these mechanisms were not measured in the present study, future research incorporating perfectionism, cognitive control, impulsivity, and emotion-regulation styles may clarify the pathways connecting health anxiety with orthorexia and eating-related psychopathology.

### 5.1 Strengths and limitations

Because our study was conducted in major cities, it is inappropriate to generalize to the entire country. Additionally, the cultural characteristics of the Turkish context, where food has strong social and emotional meaning and appearance norms are more publicly emphasized, may influence how healthy eating behaviors are expressed. Therefore, cultural specificity should be considered when interpreting the findings. Furthermore, the cross-sectional nature of this study, rather than its longitudinal nature, is a limitation. This design does not allow for the establishment of a causal relationship between the variables. Future studies employing longitudinal or experimental designs could more accurately determine the temporal sequence of ON, health anxiety and clinical eating disorder symptoms. ON, clinical eating disorders, and health anxiety can be influenced by psychological and demographic factors, and the short timeframe for clarifying these differences and their long-term effects is another limitation. Another limitation concerns the diagnostic controversies surrounding the ORTO-R. Recent literature has questioned its construct validity and its ability to distinguish clearly between adaptive, health-oriented eating patterns (“healthy orthorexia”) and maladaptive, impairment-related orthorexic behaviors (Gkiouleka et al., 2022). As orthorexia is increasingly understood as a heterogeneous phenotype, elevated ORTO-R scores in our sample may reflect a combination of these distinct dimensions, which should be considered when interpreting the findings. Furthermore, variables such as depression, perfectionism and mindfulness, which are known to affect both anxiety and eating behavior, were not measured and may act as potential confounders, influencing the observed relationships. Varying diagnostic criteria make it difficult to assess associations. According to traditional Turkish practices, most men are reluctant to answer questions in these types of surveys. Therefore, the number of men is lower than the number of women. Additionally, as all measures were based on self-report questionnaires, there is a possibility of response bias and social desirability effects, particularly with regard to sensitive behaviors such as eating patterns and anxiety levels. The unequal representation of male and female volunteers among the participants may represent a selection bias, preventing our results from being generalized to the entire population, gender, age group, and education level.

In this study, adjusted *R*^2^ values rather than eta-squared were used to evaluate model fit. This indicates that additional factors may influence the observed associations. Moreover, significant demographic differences in variables such as age, sex, and marital status suggest that these characteristics may act as potential confounders in the model. Future research would benefit from the use of interaction terms or multilevel models to better account for demographic variability.

## 6 Conclusion

Health anxiety plays a decisive role in eating behaviors, shaping food choices and negatively impacting individuals’ quality of life. This situation points to a multidimensional risk environment that can threaten not only individuals’ physical health but also their psychological well-being. This study demonstrates a significant relationship between ON, clinical eating disorders, and health anxiety. The positive correlations observed between variables suggest that individuals’ excessive preoccupation with healthy eating may increase with health anxiety. Our results support the notion that ON is not merely an obsession with healthy eating but is instead associated with eating-disorder–related symptoms. Our study found that CIA and SHAI-18 scores were higher in women than in men, suggesting a greater association with anxiety, low self-esteem, and other eating disorder symptoms in women. Consequently, the current study found health anxiety, obsession with healthy eating, and clinical eating disorders to be risk factors. Health anxiety may be associated with individuals’ food choices and with higher levels of eating-disorder–related symptoms, both of which are linked to lower quality of life. Further studies examining the role of various variables in conjunction with health anxiety will be an important step toward a more comprehensive understanding of the causes and consequences of ON and other eating disorders. In future research, longitudinal or intervention-based designs could be used to clarify the causal relationships between these variables and observe how these patterns evolve over time. Additionally, cross-cultural and experimental studies could contribute to determining whether these relationships are universal or influenced by cultural contexts.

## Data Availability

The original contributions presented in the study are included in the article/supplementary material, further inquiries can be directed to the corresponding author.

## References

[ref1] Abdullah M. A. Al Hourani H. M. Alkhatib B. (2020). Prevalence of orthorexia nervosa among nutrition students and nutritionists: pilot study. Clin Nutr. ESPEN 40, 144–148. doi: 10.1016/j.clnesp.2020.09.175, 33183528

[ref2] Albery I. P. Shove E. Bartlett G. Frings D. Spada M. M. (2022). Individual differences in selective attentional bias for healthy and unhealthy food-related stimuli and social identity as a vegan/vegetarian dissociate “healthy” and “unhealthy” orthorexia nervosa. Appetite 178:106261. doi: 10.1016/j.appet.2022.106261, 35931214

[ref3] Alshayea A. K. (2020). Latent structure, measurement invariance, and reliability of an Arabic version of the short health anxiety inventory. J. Exp. Psychopathol. 11:2043808720912629. doi: 10.1177/2043808720912629

[ref4] Argyrides M. Anastasiades E. Alexiou E. (2020). Risk and protective factors of disordered eating in adolescents based on gender and body mass index. Int. J. Environ. Res. Public Health 17:9238. doi: 10.3390/ijerph17249238, 33321884 PMC7763165

[ref5] Arnáez S. García-Soriano G. López-Santiago J. Belloch A. (2019). The Spanish validation of the short health anxiety inventory: psychometric properties and clinical utility. Int. J. Clin. Health Psychol. 19, 251–260. doi: 10.1016/j.ijchp.2019.05.003, 31516503 PMC6732766

[ref6] Atchison A. E. Zickgraf H. F. (2022). Orthorexia nervosa and eating disorder behaviors: a systematic review of the literature. Appetite 177:106134. doi: 10.1016/j.appet.2022.106134, 35750289

[ref7] Awad E. Salameh P. Sacre H. Malaeb D. Hallit S. Obeid S. (2021). Association between impulsivity and orthorexia nervosa/healthy orthorexia: any mediating effect of depression, anxiety, and stress? BMC Psychiatry 21:604. doi: 10.1186/s12888-021-03594-4, 34861836 PMC8640965

[ref8] Aydemir O. Kirpinar I. Sati T. Uykur B. Cengisiz C. (2013). Reliability and validity of the Turkish version of the health anxiety inventory. Noro Psikiyatr. Ars. 50, 325–331. doi: 10.4274/npa.y6383, 28360565 PMC5363424

[ref9] Barlow I. U. Lee E. Saling L. (2024). Orthorexia nervosa versus healthy orthorexia: anxiety, perfectionism, and mindfulness as risk and preventative factors of distress. Eur. Eat. Disord. Rev. 32, 130–147. doi: 10.1002/erv.3032, 37670425

[ref10] Barthels F. Horn S. Pietrowsky R. (2021). Orthorexic eating behaviour, illness anxiety and dysfunctional cognitions characteristic of somatic symptom disorders in a non-clinical sample. Eat. Weight Disord. 26, 2387–2391. doi: 10.1007/s40519-020-01091-3, 33392953

[ref11] Bohn K. Doll H. A. Cooper Z. O'Connor M. Palmer R. L. Fairburn C. G. (2008). The measurement of impairment due to eating disorder psychopathology. Behav. Res. Ther. 46, 1105–1110. doi: 10.1016/j.brat.2008.06.012, 18710699 PMC2764385

[ref12] Bohn K. Fairburn C. G. (2008). “The Clinical Impairment Assessment Questionnaire (CIA)” in Cognitive behavioral therapy for eating disorders. ed. Fairburn C. G. (New York: Guilford Press), 315–317.

[ref13] Cena H. Barthels F. Cuzzolaro M. Bratman S. Brytek-Matera A. Dunn T. . (2019). Definition and diagnostic criteria for orthorexia nervosa: a narrative review of the literature. Eat. Weight Disord. 24, 209–246. doi: 10.1007/s40519-018-0606-y, 30414078

[ref14] Chace S. Kluck A. S. (2021). Validation of the Teruel orthorexia scale and relationship to health anxiety in a U.S. sample. Eat. Weight Disord. 27, 1437–1447. doi: 10.1007/s40519-021-01272-8, 34379313

[ref15] Chard C. A. Hilzendegen C. Barthels F. Stroebele-Benschop N. (2019). Psychometric evaluation of the English version of the Düsseldorf Orthorexie scale (DOS) and the prevalence of orthorexia nervosa among a US student sample. Eat. Weight Disord. 24, 275–281. doi: 10.1007/s40519-018-0507-4 30196526

[ref16] Cifuentes L. Campos A. Silgado M. L. Kelpin S. Stutzman J. Hurtado M. D. . (2022). Association between anxiety and eating behaviors in patients with obesity. Obes. Pillars 3:100021. doi: 10.1016/j.obpill.2022.100021, 37990724 PMC10662093

[ref17] Cohen J. Statistical power analysis for the behavioral sciences. New York (1988).

[ref18] de Vos J. A. LaMarre A. Radstaak M. Bijkerk C. A. Bohlmeijer E. T. Westerhof G. J. (2017). Identifying fundamental criteria for eating disorder recovery: a systematic review and qualitative meta-analysis. J. Eat. Disord. 5:34. doi: 10.1186/s40337-017-0158-2 29118983 PMC5664841

[ref19] Donini L. M. Marsili D. Graziani M. P. Imbriale M. Cannella C. (2005). Orthorexia nervosa: validation of a diagnosis questionnaire. Eat. Weight Disord. 10, e28–e32. doi: 10.1007/BF03327537, 16682853

[ref20] Donini L. M. Marsili D. Graziani M. P. Imbriale M. Cannella C. (2022). Orthorexia nervosa: a comprehensive review. Eat. Weight Disord. 27, 2985–3000. doi: 10.1007/s40519-021-01312-z35247179

[ref21] Dudzikowska M. Rębak D. Gotlib-Małkowska J. Chmielewski J. Sierpiński R. Goździewska M. . (2025). Effect of overweight and obesity on clinical course and prognosis in patients with acute coronary syndromes. Ann. Agric. Environ. Med. 1–8. doi: 10.26444/aaem/21389440159730

[ref22] Dunn T. M. Bratman S. (2016). On orthorexia nervosa: a review of the literature and proposed diagnostic criteria. Eat. Behav. 21, 11–17. doi: 10.1016/j.eatbeh.2016.12.006 26724459

[ref23] Duradoni M. Gursesli M. C. Fiorenza M. Guazzini A. (2023). The relationship between orthorexia nervosa and obsessive compulsive disorder. Eur. J. Investig. Health Psychol. Educ. 13, 861–869. doi: 10.3390/ejihpe13050065, 37232703 PMC10216926

[ref24] Escolar-Llamazares M. C. Martínez-Martín M. Á. Medina-Gómez M. B. González-Alonso M. Y. Mercado-Val E. Lara-Ortega F. (2023). Sociodemographic variables and body mass index associated with the risk of eating disorders in Spanish university students. Eur. J. Investig. Health Psychol. Educ. 13, 595–612. doi: 10.3390/ejihpe13030046, 36975398 PMC10047306

[ref25] Gkiouleka M. Stavraki C. Sergentanis T. N. Vassilakou T. V. (2022). Orthorexia nervosa in adolescents and young adults: a literature review. Children 9:365. doi: 10.3390/children9030365, 35327737 PMC8947656

[ref26] Gleaves D. H. Graham E. C. Ambwani S. (2020). Measuring “orthorexia”: development, validity, and reliability of the eating habits questionnaire. Eat. Disord. 28, 253–272. doi: 10.1080/10640266.2019.1629228

[ref27] Gramaglia C. Gambaro E. Delicato C. Marchetti M. Sarchiapone M. Ferrante D. . (2019). Orthorexia nervosa, eating patterns and personality traits: a cross-cultural comparison of Italian, polish and Spanish university students. BMC Psychiatry 19:235. doi: 10.1186/s12888-019-2208-2, 31362720 PMC6668093

[ref28] Greville-Harris M. Talbot C. V. Moseley R. L. Vuillier L. (2022). Conceptualisations of health in orthorexia nervosa: a mixed-methods study. Eat. Weight Disord. 27, 3135–3143. doi: 10.1007/s40519-022-01443-1, 35861935 PMC9301897

[ref29] Hallit S. Brytek-Matera A. Obeid S. (2021). Orthorexia nervosa and disordered eating attitudes among Lebanese adults: assessing psychometric proprieties of the ORTO-R in a population-based sample. PLoS One 16:e0254948. doi: 10.1371/journal.pone.0254948, 34437545 PMC8389519

[ref30] He J. Brytek-Matera A. Cooper M. Cui S. Chen G. (2022). Chinese translation of the clinical impairment assessment (CIA 3.0): psychometric properties and measurement invariance across sex and age in adolescents, young adults, and adult men. Eat. Behav. 45:101623. doi: 10.1016/j.eatbeh.2022.101623, 35303545

[ref31] Jahrami H. Saif Z. Faris M. A. Levine M. P. (2019). The relationship between risk of eating disorders, age, gender and body mass index in medical students: a meta-regression. Eat. Weight Disord. 24, 169–177. doi: 10.1007/s40519-018-0618-7, 30430465

[ref32] Kris-Etherton P. M. Petersen K. S. Hibbeln J. R. Hurley D. Kolick V. Peoples S. . (2021). Nutrition and behavioral health disorders: depression and anxiety. Nutr. Rev. 79, 247–260. doi: 10.1093/nutrit/nuaa103 32447382 PMC8453603

[ref33] Levin R. L. Mills J. S. McComb S. E. Rawana J. S. (2023). Examining orthorexia nervosa: using latent profile analysis to explore potential diagnostic classification and subtypes in a non-clinical sample. Appetite 181:106398. doi: 10.1016/j.appet.2022.106398, 36455786

[ref34] Łucka I. Mazur A. Łucka A. Sarzyńska I. Trojniak J. Kopańska M. (2024). Orthorexia as an eating disorder spectrum-a review of the literature. Nutrients 16:3304. doi: 10.3390/nu16193304, 39408271 PMC11478848

[ref35] Łucka I. Mazur A. Łucka A. Trojniak J. Kopańska M. (2025). Orthorexia nervosa tendencies in two cohorts of polish young adults: a comparative analysis of prevalence, correlates, and comorbidity. Nutrients 17:2208. doi: 10.3390/nu17132208, 40647311 PMC12252250

[ref36] Luck-Sikorski C. Jung F. Schlosser K. Riedel-Heller S. G. (2019). Is orthorexic behavior common in the general public? A large representative study in Germany. Eat. Weight Disord. 24, 267–273. doi: 10.1007/s40519-018-0502-5, 29564745

[ref37] McComb S. E. Mills J. S. (2019). Orthorexia nervosa: a review of psychosocial risk factors. Appetite 140, 50–75. doi: 10.1016/j.appet.2019.05.005, 31075324

[ref38] Meier M. Kossakowski J. J. Jones P. J. Kay B. Riemann B. C. McNally R. J. (2020). Obsessive-compulsive symptoms in eating disorders: a network investigation. Int. J. Eat. Disord. 53, 362–371. doi: 10.1002/eat.23205 31749199

[ref39] Mitrofanova E. Mulrooney H. Pummell E. Petróczi A. (2025). Tracking the shift from health to harm: development and validation of a short screening tool for orthorexia nervosa (STONE). Appetite 214:108227. doi: 10.1016/j.appet.2025.108227, 40659218

[ref40] Mosewich A. D. Spencer H. M. Kowalski K. C. (2022). Self-compassion, body image, and eating attitudes in women: examining moderated mediation. Body Image 41, 280–289. doi: 10.1016/j.bodyim.2022.01.005

[ref41] Myrick J. G. Willoughby J. F. (2019). Educated but anxious: how emotional states and education levels combine to influence online health information seeking. Health Inform. J. 25, 649–660. doi: 10.1177/1460458219839613 28728457

[ref42] Novara C. Maggio E. Piasentin S. Pardini S. Mattioli S. (2021a). Orthorexia nervosa: differences between clinical and non-clinical samples. BMC Psychiatry 21:341. doi: 10.1186/s12888-021-03348-2, 34238282 PMC8265101

[ref43] Novara C. Pardini S. Maggio E. Mattioli S. Piasentin S. (2021b). Orthorexia nervosa: over concern or obsession about healthy food? Eat. Weight Disord. 26, 2577–2588. doi: 10.1007/s40519-021-01110-x, 33566324 PMC8602217

[ref44] Oberle C. D. Samaghabadi R. O. Hughes E. M. (2017). Orthorexia nervosa: assessment and correlates with gender, BMI, and personality. Appetite 108, 303–310. doi: 10.1016/j.appet.2016.10.021, 27756637

[ref45] Pontillo M. Zanna V. Demaria F. Averna R. Di Vincenzo C. Di Biase M. . (2022). Orthorexia nervosa, eating disorders, and obsessive-compulsive disorder: a selective review of the last seven years. J. Clin. Med. 11:6134. doi: 10.3390/jcm11206134, 36294454 PMC9604819

[ref46] Reas D. L. Rø Ø. Kapstad H. Lask B. (2010). Psychometric properties of the clinical impairment assessment: norms for young adult women. Int. J. Eat. Disord. 43, 72–76. doi: 10.1002/eat.20676 19260038

[ref47] Reivan Ortiz G. G. Elizalde B. Tapia C. Granero R. (2025). Psychoneurological links contributing to body mass index and eating disorder severity. Nutrients 17:296. doi: 10.3390/nu17020296, 39861426 PMC11767959

[ref48] Reynolds R. (2018). Is the prevalence of orthorexia nervosa in an Australian university population 6.5%? Eat. Weight Disord. 23, 453–458. doi: 10.1007/s40519-017-0402-0 29956098

[ref49] Rogoza R. Donini L. M. (2021). Introducing ORTO-R: a revision of ORTO-15 based on the re-assessment of original data. Eat. Weight Disord. 26, 887–895. doi: 10.1007/s40519-020-00924-5, 32436165 PMC8004519

[ref50] Rossi A. A. Mannarini S. Donini L. M. Castelnuovo G. Simpson S. Pietrabissa G. (2024). Dieting, obsessive-compulsive thoughts, and orthorexia nervosa: assessing the mediating role of worries about food through a structural equation model approach. Appetite 193:107164. doi: 10.1016/j.appet.2023.107164, 38103790

[ref51] Sahlan R. N. Saunders J. F. Perez M. Blomquist K. K. Fitzsimmons-Craft E. E. Bodell L. P. (2022). The validation of a Farsi version of the clinical impairment assessment (F-CIA) among Iranian adolescent boys and girls. Eat. Weight Disord. 27, 665–674. doi: 10.1007/s40519-021-01199-1 33970468

[ref52] Salkovskis P. M. Rimes K. A. Warwick H. M. C. Clark D. M. (2002). The health anxiety inventory: development and validation of scales for the measurement of health anxiety and hypochondriasis. Psychol. Med. 32, 843–853. doi: 10.1017/S0033291702005822, 12171378

[ref53] Sanlier N. Yassibas E. Bilici S. Sahin G. Celik B. (2016). Does the rise in eating disorders lead to increasing risk of orthorexia nervosa? Correlations with gender, education, and body mass index. Ecol. Food Nutr. 55, 266–278. doi: 10.1080/03670244.2016.1150276, 26979290

[ref54] Sanseverino R. Guidotti S. Pruneti C. (2025). Assessing orthorexia nervosa among university students: an observational study analyzing prevalence and psychological characteristics. Nutrients 17:2078. doi: 10.3390/nu17132078, 40647183 PMC12251277

[ref55] Sirri L. Tossani E. Potena L. Masetti M. Grandi S. (2020). Manifestations of health anxiety in patients with heart transplant. Heart Lung 49, 364–369. doi: 10.1016/j.hrtlng.2019.12.003 32145959

[ref56] Strahler J. (2020). The dark side of healthy eating: links between orthorexic eating and mental health. Nutrients 12:3662. doi: 10.3390/nu12123662, 33260760 PMC7761061

[ref57] Strahler J. (2021). Orthorexia nervosa: a review of psychosocial correlates and new directions for treatment research. Appetite 160:105082. doi: 10.1016/j.appet.2021.105082

[ref58] Strahler J. Hermann A. Walter B. Stark R. (2018). Orthorexia nervosa: a behavioral complex or a psychological condition? J. Behav. Addict. 7, 1143–1156. doi: 10.1556/2006.7.2018.129, 30556782 PMC6376377

[ref59] Strahler J. Wachten H. Neuhofer S. Zimmermann P. (2022). Psychological correlates of excessive healthy and orthorexic eating: emotion regulation, attachment, and anxious-depressive-stress symptomatology. Front. Nutr. 9:817047. doi: 10.3389/fnut.2022.817047, 35356731 PMC8959669

[ref60] Tan E. J. Raut T. Le L. K. Hay P. Ananthapavan J. Lee Y. Y. . (2023). The association between eating disorders and mental health: an umbrella review. J. Eat. Disord. 11:51. doi: 10.1186/s40337-022-00725-4, 36973817 PMC10044389

[ref61] Vaz A. R. Conceição E. Pinto-Bastos A. Silva D. Machado P. P. (2020). Validation of the Portuguese version of the clinical impairment assessment (CIA) in eating disorders’ patients. Eat. Weight Disord. 25, 627–635. doi: 10.1007/s40519-019-00755-x 30838511

[ref62] Witaszek T. Babicki M. Brytek-Matera A. Mastalerz-Migas A. Kujawa K. Kłoda K. (2023). Maladaptive eating behaviours, generalised anxiety disorder and depression severity: a comparative study between adult women with overweight, obesity, and normal body mass index range. Nutrients 16:80. doi: 10.3390/nu16010080, 38201910 PMC10780963

[ref63] World Health Organization. Body mass index. WHO. (2025). Available online at: https://www.euro.who.int/en/health-topics/disease-prevention/nutrition/ahealthy-lifestyle/body-mass-index-bmi (Accessed June 16, 2025).

[ref64] Yılmaz M. N. Dundar C. (2022). The relationship between orthorexia nervosa, anxiety, and self-esteem: a cross-sectional study in Turkish faculty members. BMC Psychol. 10:82. doi: 10.1186/s40359-022-00796-7, 35361269 PMC8974066

[ref65] Zagaria A. Barbaranelli C. Mocini E. Lombardo C. (2023). Cross-cultural adaptation and psychometric properties of the Italian version of the orthorexia nervosa inventory (ONI). J. Eat. Disord. 11:144. doi: 10.1186/s40337-023-00858-0, 37620907 PMC10463941

[ref66] Zagaria A. Vacca M. Cerolini S. Ballesio A. Lombardo C. (2022). Associations between orthorexia, disordered eating, and obsessive-compulsive symptoms: a systematic review and meta-analysis. Int. J. Eat. Disord. 55, 295–312. doi: 10.1002/eat.23654, 34921564

[ref67] Zickgraf H. F. Barrada J. R. (2022). Orthorexia nervosa vs. healthy orthorexia: relationships with disordered eating, eating behavior, and healthy lifestyle choices. Eat. Weight Disord. 27, 1313–1325. doi: 10.1007/s40519-021-01263-9, 34275120 PMC8286169

[ref68] Zickgraf H. F. Ellis J. M. Essayli J. H. (2019). Disentangling orthorexia nervosa from healthy eating and other eating disorder symptoms: relationships with clinical impairment, comorbidity, and self-reported food choices. Appetite 134, 40–49. doi: 10.1016/j.appet.2018.12.006, 30543837 PMC8056745

